# The causal association between genetically regulated 25OHD and chronic obstructive pulmonary disease: A meta-analysis and Mendelian randomization study

**DOI:** 10.3389/fgene.2022.932764

**Published:** 2022-10-19

**Authors:** Min Yang, Bo Pang, Qiong Wang, Zhixin Zhang, Wenquan Niu

**Affiliations:** ^1^ Department of Acupuncture and Moxibustion, Guang’anmen Hospital, China Academy of Chinese Medical Sciences, Beijing, China; ^2^ Graduate School, Beijing University of Chinese Medicine, Beijing, China; ^3^ Department of Pediatrics, China-Japan Friendship Hospital, Beijing, China; ^4^ International Medical Services, China-Japan Friendship Hospital, Beijing, China; ^5^ Institute of Clinical Medical Sciences, China-Japan Friendship Hospital, Beijing, China

**Keywords:** chronic obstructive pulmonary disease, vitamin D binding protein, polymorphism, meta-analysis, Mendelian randomization

## Abstract

**Backgrounds and objectives:** Chronic obstructive pulmonary disease (COPD) is a multifactorial disease under genetic control. We present a meta-analysis to examine the associations of vitamin D binding protein (*VDBP*) gene rs7041 polymorphism with the risk of COPD and changes in circulating 25OHD concentrations.

**Methods:** A literature search, quality assessment, and data extraction were conducted independently by two investigators. Data are expressed as odds ratio (OR) or weighted mean difference (WMD) with a 95% confidence interval (CI). The inverse variance weighted method (IVW) in R (version 1.1.456) was applied to calculate the Mendelian randomization coefficient.

**Results:** A total of 13 articles with 3,667 participants were meta-analyzed. The rs7041-GT genotype was associated with a 49% reduced COPD risk (OR: 0.51, 95% CI: 0.30 to 0.88, *p* = 0.014) compared to the rs7041-TT genotype. Carriers of the rs7041-GT genotype had significantly higher concentrations of circulating 25OHD than those with the rs7041-TT genotype (WMD: 0.32 ng/ml, 95% CI: 0.09 to 0.55, *p* = 0.006). Under the assumptions of Mendelian randomization, and assuming a linear logistic relationship between circulating 25OHD and COPD, an inverse association was noted after using *VDBP* gene rs7041 polymorphism as an instrument (WMD: −2.07, 95% CI: −3.72 to −0.41, *p* = 0.015). There was a low probability of publication bias.

**Conclusion:** We observed significant associations of *VDBP* gene rs7041 polymorphism with the risk of COPD and changes in circulating 25OHD concentrations. Importantly, we found a causal relationship between genetically regulated 25OHD concentrations and COPD risk.

## Introduction

Chronic obstructive pulmonary disease (COPD) places a major burden on individuals and public health systems (Cho et al., 2022). The Global Burden of Disease study estimated that 3.2 million people died from COPD in 2015 worldwide, representing an increase of 11.6% compared with 1990 (GBD 2015 Chronic Respiratory Disease Collaborators, 2017). In China, the overall prevalence of spirometry-defined COPD was 8.6%, accounting for nearly 100 million patients with COPD (Wang et al., 2018). COPD prevention should be an urgent strategy that can be implemented by determining potential risk factors and identifying persons who are at risk of developing COPD and who could be targeted for preventive measures.

COPD is a multifactorial disease under genetic control (Marsh et al., 2006; Agustí et al., 2022). The heritability of COPD has been estimated to be 37.7% (Zhou et al., 2013), which emphasizes a clear rationale for the determination of COPD-susceptibility genes or genetic alterations (Silverman et al., 2011; Silverman, 2020). Dozens of genome-wide association studies have been undertaken to decipher the genetic linings of COPD (Pillai et al., 2009; Hansel et al., 2015; Wain et al., 2017); however, one of the major challenges in investigating genetic determinants is heterogeneity. Currently, the list of candidate genes for COPD is constantly being improved and updated. One of the most widely evaluated genes is the gene coding for the vitamin D binding protein (*VDBP*).

The human *VDBP* gene is localized in chromosome 4q11-q13; it has three commonly recognized haplotypes (GC-1F, GC-1S, and GC-2) and rs7041 polymorphisms are one of the most common non-synonymous single-nucleotide polymorphisms (SNPs) for the haplotypes (Cleve and Constans, 1988). The three haplotypes have been reported to have a diverging affinity to 25OHD, the marker that is the best indicator of circulating vitamin D concentrations (Arnaud and Constans, 1993). In addition, *VDBP* genetic alterations have been found to be associated with vitamin D deficiency (Chishimba et al., 2010; Wood et al., 2011). The susceptibility of *VDBP* haplotypes to COPD risk has been widely evaluated. A meta-analysis by Khanna et al. (2019) showed that the GC-1F haplotype and the GC-1F/1F genotype in the *VDBP* gene impose a significant genetic risk for COPD among Asians. In another meta-analysis by Xie et al. (2015), it was found that the GC-1F homozygote may be a risk-conferring factor for COPD and that the GC-2 homozygote may be a protective factor against COPD. To the best of our knowledge, no pooled evidence exists currently on rs7041 polymorphism in the *VDBP* gene associated with COPD. At present, this association is the subject of much debate. For example, Li et al. (2014) found that homozygous carriers of the rs7041 T allele had enhanced susceptibility to COPD, whereas Ishii et al. (2014) failed to detect any significant link between rs7041 and COPD. The causes of these inconsistent findings could be explored by a comprehensive synthesis of published association studies.

To seek the possible causes and offer research insights, we present a meta-analysis to examine the associations of *VDBP* gene rs7041 polymorphism with the risk of COPD and changes in circulating 25OHD concentrations. In the case of statistical significance being found for both associations, we attempt to explore the possibly causal implications of circulating 25OHD in the development of COPD by adopting the Mendelian randomization technique and using rs7041 polymorphism as an instrument.

## Methods

This meta-analysis was conducted followed the guidelines in the preferred reporting items for systematic reviews and meta-analyses (PRISMA) statement (Moher et al., 2009). The PRISMA checklist is provided in Supplementary Table S1.

### Search strategy

A literature search was carried out in the three online databases (PubMed, EMBASE, and Web of Science) up March 10, 2022, including articles ahead of publication. The following keywords were used in searching: (“Vitamin D binding protein” OR “VDBP” OR “VDB” OR “DBP” OR “Gc-globulin” OR “Gc-globulin” OR “GC”) AND (“COPD” OR “chronic obstructive pulmonary disease”) AND (“polymorphism” OR “SNP” OR “variant” OR “variation” OR “mutation” OR “single nucleotide polymorphisms”).

Two authors (MY and BP) completed the literature search independently, and any disagreement was solved by discussion. In addition, the references of major reviews or meta-analyses were also checked for potentially eligible articles that were not identified by the two authors.

### Inclusion criteria

Articles eligible for inclusion in this meta-analysis must simultaneously meet the following criteria: 1) case-control design; 2) published in the English language; 3) providing genotype counts of *VDBP* gene rs7041 polymorphism in COPD patients and controls or mean or median values of circulating vitamin D concentrations across rs7041 genotypes; 4) a clear definition of COPD [COPD is diagnosed by doctors or according to the Global Initiative for Chronic Obstructive Lung Disease (GOLD) or American Thoracic Society (ATS) guidelines]; and 5) a validated genotype assaying method. Moreover, in cases when more than one article was published using the same or part of the same sample of study participants, the article using the largest sample size was included.

### Exclusion criteria

Articles were excluded if any one of the following criteria was met: 1) published in form of a review, correspondence, comment, conference abstract, case report, case series, or clinical trial; 2) a clinical outcome other than COPD; and 3) the lack of a control group.

### Data extraction

Data were extracted from each eligible article independently by two authors (MY and BP) according to a predefined template, covering the surname of the first author, year of publication, country where the study was performed, ethnicity, sample size, source of controls, matched condition, diagnosis criteria of COPD, genotype counts of rs7041 polymorphism between patients and controls, mean or median 25OHD concentrations across rs7041 genotypes in patients or controls or both, and baseline characteristics of study participants including age, gender, body mass index (BMI), percentage of smokers, smoking exposure (cigarette pack-years), smoking status, forced expiratory volume in 1 s (FEV_1_), FEV_1_ predicted ratio, forced vital capacity (FVC), FVC predicted ratio, and FEV_1_/FVC, if available.

### Statistical analyses

STATA software version 14.1 (StataCorp, College Station, TX, United States) was utilized in this meta-analysis. The weighted odds ratio (OR) and the 95% confidence interval (CI) were calculated to quantify the association of *VDBP* gene rs7041 polymorphism with the risk of COPD. The weighted mean difference (WMD) and 95% CI were calculated to quantify the changes of circulating 25OHD concentrations between genotypes of this polymorphism in COPD patients or controls or both (*p* < 0.05; statistically significant). The Hardy–Weinberg equilibrium for *VDBP* gene rs7041 polymorphism was calculated using the R package “GWASExactHW” (version 1.1.456), and *p* > 0.05 indicated no deviation from the Hardy–Weinberg equilibrium.

The fraction of variation owing to heterogeneity was estimated using *I*-squared (*I* ^*2*^). A larger *I* ^*2*^ value is indicative of a higher probability of between-study heterogeneity. Significance for *I* ^*2*^ was set at 50% (Higgins et al., 2003). Whatever the magnitude of heterogeneity, the random-effects model was used to calculate effect–size estimates. The causes for between-study heterogeneity were explored from clinical and methodological aspects by subgroup analyses and meta-regression analyses.

Cumulative analyses were performed to evaluate the influence of the first publication on the association between *VDBP* gene rs7041 polymorphism and the risk of COPD on subsequent publications on the same subject over time. In addition, sensitivity analyses were performed to evaluate the contribution of single publications to pooled effect-size estimates by sequentially omitting one publication each time and deriving estimates from the remaining publications.

Publication bias was evaluated using Begg’s funnel plots and Egger’s regression asymmetry tests. The Egger test can detect funnel plot asymmetry by quantifying the probability of publication bias at a significance level of 10%. In addition, the trim-and-fill method was used to estimate the number of potentially missing publications leading to publication bias and to derive corrected pooled estimates.

The Mendelian randomization technique was employed to estimate the causal influencer of a modifiable risk factor from observational data on clinical outcomes (Smith and Ebrahim, 2003). The inverse variance weighted method (IVW) (Burgess et al., 2013) in the R programming environment (version 1.1.456) was applied to calculate the Mendelian randomization coefficient in this meta-analysis.

## Results

### Eligible studies

**FIGURE 1:**
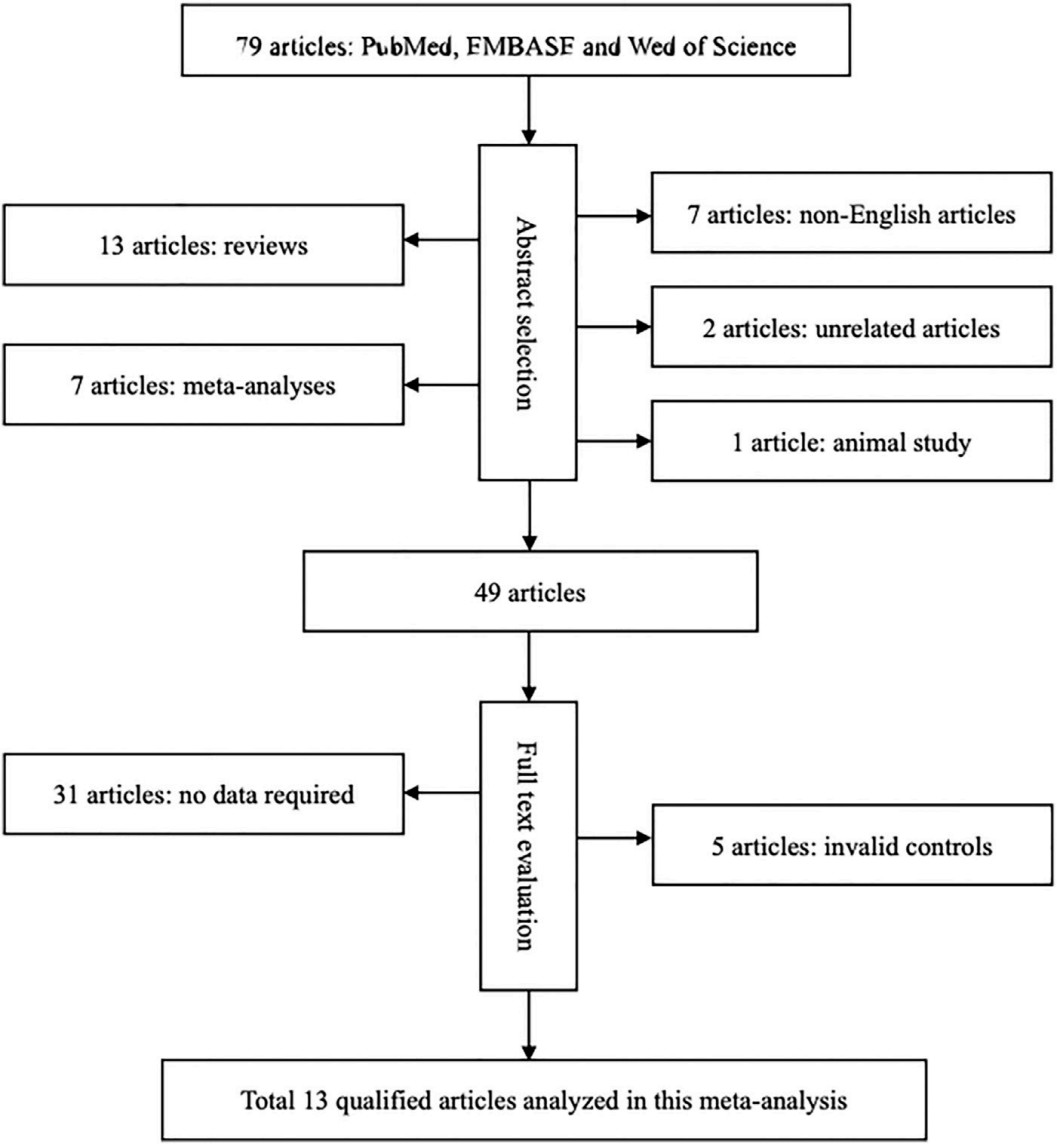
Flow diagram for the selection of qualified articles.

The initial search yielded 79 potentially relevant publications. Application of the inclusion and exclusion criteria revealed 13 eligible articles involving 3,389 participants in the final analysis (Horne et al., 1990; Ishii et al., 2001; Ito et al., 2004; Laufs et al., 2004; Janssens et al., 2010; Shen et al., 2010; Ishii et al., 2014; Jung et al., 2014; Maheswari et al., 2014; Al-Azzawi et al., 2017; Chuaychoo et al., 2018; Jolliffe et al., 2018; Gao et al., 2020). The selection process with specific reasons for exclusion is presented in Figure 1. Finally, data from the 3,667 participants were pooled. Data on circulating 25OHD changes across the rs7041 genotypes were extracted from two articles involving 540 patients and 152 controls (Janssens et al., 2010; Jolliffe et al., 2018).

### Study characteristics

The baseline characteristics of the study populations are summarized in Table 1. Of the 13 eligible articles, seven were performed among Asians (Ishii et al., 2001; Ito et al., 2004; Shen et al., 2010; Ishii et al., 2014; Jung et al., 2014; Maheswari et al., 2014; Chuaychoo et al., 2018), four among Caucasians (Horne et al., 1990; Laufs et al., 2004; Janssens et al., 2010; Gao et al., 2020), one among a Middle Eastern population (Maheswari et al., 2014), and one among a mixed population (Jolliffe et al., 2018). Total sample sizes ranged from 100 to 517. A total of seven articles were conducted with matched patients and controls. There was no deviation from the Hardy–Weinberg equilibrium for *VDBP* gene rs7041 polymorphism. Table 1 also shows the quality assessment of all the qualified articles by using the Newcastle–Ottawa scale (NOS) tool for cohort studies.

**TABLE 1:** Baseline characteristics of all involved studies in this meta-analysis.

First author	Year	Country	Ethnicity	Sample size	Patients	Controls	Match	Diagnosis	COPD stage	Source of controls	NOS score	Age (years)	
												Patients	Controls
Horne	1990	Canada	Caucasian	517	104	413	Yes	Doctor	NA	Population	9	NA	NA
Ishii	2001	Japan	Asian	145	63	82	Yes	ATS	NA	Hospital	8	68.3 (9.9)	NA
Laufs	2004	Iceland	Caucasian	285	102	183	No	ATS	NA	Population	8	71.1	42.9
Ito	2004	Japan	Asian	191	103	88	Yes	ATS	NA	Hospital	8	67.4 (7.8)	60.8 (12.0)
Shen	2010	China	Asian	200	100	100	No	ATS	NA	Hospital	7	62.3 (9.7)	60.9 (8.6)
Janssens	2010	Belgium	Caucasian	414	262	152	Yes	ATS	I-IV	Population	9	66 (60–72)	61 (58–65)
Jung	2014	South Korea	Asian	360	203	157	No	ATS	I-IV	Population	8	67 (9.03)	53 (8.89)
Kukkonen	2014	India	Asian	100	50	50	Yes	Doctor	I-IV	Hospital	8	55.86 (7.42)	55.82 (7.89)
Ishii	2014	Japan	Asian	580	361	219	No	GOLD	I-IV	Hospital	7	68.9 (8.5)	63.1 (12.0)
Al-Azzawi	2017	Egypt	Middle Eastern	160	80	80	Yes	ATS	I-IV	Hospital	8	55.0 (9.0)	53.1 (7.4)
Al-Azzawi (Smokers)	2017	Egypt	Middle Eastern	120	80	40	Yes	ATS	I-IV	Hospital	8	55.0 (9.0)	52.1 (6.5)
Al-Azzawi (Non-smokers)	2017	Egypt	Middle Eastern	120	80	40	Yes	ATS	I-IV	Hospital	8	55.0 (9.0)	55.7 (8.5)
Al-Azzawi (GOLD I)	2017	Egypt	Middle Eastern	96	16	80	Yes	ATS	I	Hospital	8	NA	53.05 (7.4)
Al-Azzawi (GOLD II)	2017	Egypt	Middle Eastern	120	40	80	Yes	ATS	II	Hospital	8	NA	53.05 (7.4)
Al-Azzawi (GOLD III-IV)	2017	Egypt	Middle Eastern	104	24	80	Yes	ATS	III-IV	Hospital	8	NA	53.05 (7.4)
Jolliffe	2018	United Kingdom	Mixed	269	269	0	NA	Doctor	I-IV	NA	7	66.4 (9.5)	NA
Chuaychoo	2018	Thailand	Asian	204	136	68	No	GOLD	NA	Hospital	7	71.0 (8.8)	65.5 (8.9)
Gao	2020	Finland	Caucasian	233	44	189	Yes	GOLD	I-III	Population	9	58.9 (7.1)	53.5 (9.3)
Gao (Non-smokers)	2020	Finland	Caucasian	76	44	32	Yes	GOLD	I-III	Population	9	58.9 (7.1)	54.9 (9.6)
Gao (Control Smokers)	2020	Finland	Caucasian	201	44	157	Yes	GOLD	I-III	Population	9	58.9 (7.1)	52.1 (9.0)
Gao (GOLD I & Smokers)	2020	Finland	Caucasian	210	21	189	Yes	GOLD	I	Population	9	55.1 (8.3)	53.5 (9.3)
Gao (GOLD II-III & Smokers)	2020	Finland	Caucasian	212	23	189	Yes	GOLD	II-III	Population	9	62.7 (5.8)	53.5 (9.3)

Gender (males, %)		BMI (kg/m^2^)		Smokers (%)		CPY (pack-years)		FEV_1_ (L)		Predicted FEV_1_ (%)		FVC (L)		Predicted FVC (%)	
Patients	Controls	Patients	Controls	Patients	Controls	Patients	Controls	Patients	Controls	Patients	Controls	Patients	Controls	Patients	Controls
NA	NA	NA	NA	NA	NA	NA	NA	NA	NA	NA	NA	NA	NA	NA	NA
0.95	NA	NA	NA	NA	NA	102.2 (40.4)	NA	NA	NA	NA	NA	NA	NA	NA	NA
0.41	0.57	NA	NA	0.93	NA	38	NA	NA	NA	NA	NA	NA	NA	NA	NA
0.96	0.82	NA	NA	NA	NA	58.3 (29.1)	41.1	1.2 (0.5)	2.7 (0.9)	45.3 (18.4)	88.4 (20.7)	NA	NA	NA	NA
0.72	0.66	NA	NA	NA	NA	123.5 (29.7)	25.6 (13.3)	1.3 (0.3)	3.6 (0.7)	NA	NA	NA	NA	NA	NA
0.82	0.79	NA	NA	1.00	1.00	47.0 (33.0,63.0)	NA	1.8 (0.9)	3.2 (0.8)	61.0 (27.0)	104.0 (15.0)	3.4 (1.0)	4.1 (0.9)	93.0 (22.0)	110.0 (14.0)
0.98	0.94	NA	NA	1.00	1.00	46 (23.4)	30.7 (17.4)	1.4 (0.5)	3.2 (0.6)	53.1 (16.9)	93.9 (12.8)	3.0 (0.8)	4.1 (0.7)	79.6 (19.3)	93.3 (11.9)
0.76	0.76	NA	NA	0.76	NA	23.6 (18.1)	NA	NA	NA	NA	NA	NA	NA	NA	NA
0.92	0.83	NA	NA	1.00	1.00	65.7 (39.3)	51.1 (40.6)	1.7 (0.7)	2.8 (0.8)	60.3 (20.4)	95.8 (15.5)	3.2 (0.9)	3.5 (0.9)	94.6 (18)	99 (15.8)
0.73	0.78	NA	NA	0.50	0.50	46.4 (1.7)	33.7 (1.2)	NA	NA	NA	NA	NA	NA	NA	NA
NA	NA	NA	NA	0.50	1.00	46.4 (1.7)	33.7 (1.2)	NA	NA	NA	NA	NA	NA	NA	NA
NA	NA	NA	NA	0.50	0.00	46.4 (1.7)	0	NA	NA	NA	NA	NA	NA	NA	NA
NA	NA	NA	NA	NA	0.50	NA	33.7 (1.2)	NA	NA	NA	NA	NA	NA	NA	NA
NA	NA	NA	NA	NA	0.50	NA	33.7 (1.2)	NA	NA	NA	NA	NA	NA	NA	NA
NA	NA	NA	NA	NA	0.50	NA	33.7 (1.2)	NA	NA	NA	NA	NA	NA	NA	NA
1	NA	27.7 (6.8)	NA	1	NA	NA	NA	NA	NA	NA	NA	NA	NA	NA	NA
NA	NA	22.8 (3.9)	24.7 (3.7)	NA	NA	46.5 (54.1)	1.7 (3.9)	1.3 (0.5)	2.6 (0.5)	63.9 (24.6)	111.5 (18.0)	2.5 (0.6)	3.3 (0.6)	92.1 (24.5)	110.9 (21.3)
0.86	0.52	27.3 (3.9)	26.5 (3.7)	1.00	0.83	39.3 (12.9)	28.4 (14.1)	2.7 (0.5)	3.3 (0.7)	75.5 (8.3)	101.5 (13.6)	4.2 (0.8)	3.9 (0.9)	95.8 (5.7)	99.3 (12.2)
0.86	0.38	27.3 (3.9)	26.2 (3.6)	1.00	0.00	39.3 (12.9)	0	2.7 (0.5)	3.2 (0.6)	75.5 (8.3)	106.5 (14.6)	4.2 (0.8)	3.8 (0.8)	95.8 (5.7)	102.3 (12.3)
0.86	0.55	27.3 (3.9)	26.9 (3.8)	1.00	1.00	39.3 (12.9)	28.4 (14.1)	2.7 (0.5)	3.3 (0.8)	75.5 (8.3)	96.5 (12.5)	4.2 (0.8)	4.0 (1.0)	95.8 (5.7)	96.3 (12.1)
0.86	0.52	26.2 (3.3)	26.5 (3.7)	1.00	0.83	35.5 (12.7)	28.4 (14.1)	3.2 (0.5)	3.25 (0.7)	87.3 (5.2)	101.5 (13.6)	4.8 (0.8)	3.9 (0.9)	104.9 (0.1)	99.3 (12.2)
0.87	0.52	28.3 (4.4)	26.5 (3.7)	1.00	0.83	43.1 (13.0)	28.4 (14.1)	2.1 (0.5)	3.25 (0.7)	63.8 (11.4)	101.5 (13.6)	3.6 (0.7)	3.9 (0.9)	86.6 (11.2)	99.3 (12.2)

FEV_1_/FVC (%)		rs7041-GG genotype		rs7041-GT genotype		rs7041-TT genotype		25OHD across rs7041 (G/C) genotypes in patients (ng/ml)			25OHD across rs7041 (G/C) genotypes in controls (ng/ml)		
Patients	Controls	Patients	Controls	Patients	Controls	Patients	Controls	rs7041GG	rs7041GT	rs7041TT	rs7041GG	rs7041GT	rs7041TT
NA	NA	39	141	0	201	16	72	NA	NA	NA	NA	NA	NA
44.3 (12.7)	NA	20	6	0	32	12	44	NA	NA	NA	NA	NA	NA
NA	NA	38	71	0	86	16	26	NA	NA	NA	NA	NA	NA
45.8 (10.4)	80.1 (7.8)	5	6	0	34	62	47	NA	NA	NA	NA	NA	NA
43.5 (12.8)	92.8 (5.8)	7	7	39	39	54	53	NA	NA	NA	NA	NA	NA
NA	NA	85	54	108	73	60	23	22.1 (7.8)	20.0 (7.0)	16.7 (7.2)	26.3 (8.7)	24.6 (9.1)	20.9 (5.0)
47.5 (10.9)	78.3 (5.1)	9	13	68	65	125	79	NA	NA	NA	NA	NA	NA
NA	NA	8	11	0	25	17	15	NA	NA	NA	NA	NA	NA
51.6 (11.6)	78.6 (5.8)	26	15	131	78	202	125	NA	NA	NA	NA	NA	NA
59 (3.7)	NA	19	25	40	39	22	16	NA	NA	NA	NA	NA	NA
NA	NA	19	10	40	20	22	11	NA	NA	NA	NA	NA	NA
NA	NA	19	16	40	19	22	6	NA	NA	NA	NA	NA	NA
NA	NA	2	25	7	39	8	16	NA	NA	NA	NA	NA	NA
NA	NA	10	25	20	39	11	16	NA	NA	NA	NA	NA	NA
NA	NA	8	25	12	39	4	16	NA	NA	NA	NA	NA	NA
NA	NA	65	NA	147	NA	57	NA	50.3 (28.8)	44.0 (25.5)	40.9 (19.7)	NA	NA	NA
51.2 (12.3)	78.9 (11.4)	13	3	70	28	53	37	NA	NA	NA	NA	NA	NA
63.5 (5.3)	83.1 (5.4)	24	76	17	93	3	20	NA	NA	NA	NA	NA	NA
63.5 (5.3)	84.5 (5.3)	24	8	17	19	3	5	NA	NA	NA	NA	NA	NA
63.5 (5.3)	81.6 (5.4)	24	68	17	74	3	15	NA	NA	NA	NA	NA	NA
67.2 (2.7)	83.1 (5.4)	10	76	10	93	1	20	NA	NA	NA	NA	NA	NA
59.8 (7.8)	83.1 (5.4)	14	76	7	93	2	20	NA	NA	NA	NA	NA	NA

Abbreviations: COPD, chronic obstructive pulmonary disease; FEV_1_, forced expiratory volume in one second; FVC, forced vital capacity; GOLD, Global Initiative for Chronic Obstructive Lung Disease; ATS, American Thoracic Society; CPY, cigarette pack-years; NOS, Newcastle–Ottawa scale; NA, not available.

### Overall analysis: *VDBP* and COPD

**FIGURE 2:**
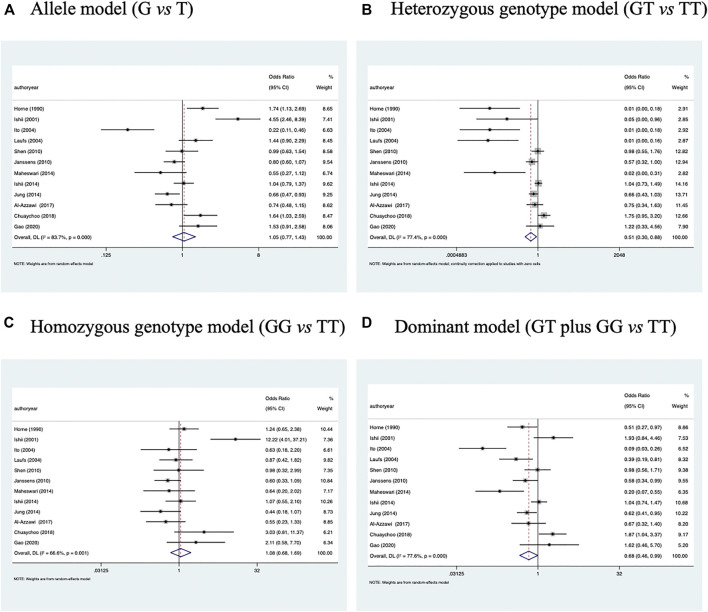
Forest plots of *VDBP* gene rs7041 polymorphism with COPD under four models of inheritance. **(A)** Allele model (G vs. T), **(B)** Heterozygous genotype model (GT vs. TT), **(C)** Homozygous genotype model (GG vs. TT), **(D)** Dominant model (GT plus GG vs. TT).

As shown in Figure 2, the association of *VDBP* gene rs7041 polymorphism with the risk of COPD was tested under four genetic models: allele model (rs7041-G vs*.* rs7041-T); homozygous genotype model (rs7041-GG vs*.* rs7041-TT); heterozygous genotype model (rs7041-GT vs*.* rs7041-TT); and dominant model (rs7041-GG + GT vs*.* rs7041-TT). This association was significant under the heterozygous genotype model and the dominant model, with the odds of having COPD being 0.51 (95% CI: 0.30 to 0.88, *p* = 0.014) and 0.68 (95% CI: 0.46 to 0.99, *p* = 0.047), respectively; however, there was moderate evidence of heterogeneity between studies for both models, with a corresponding *I* ^*2*^ value of 77.4% and 77.6%, respectively.

### Subgroup analyses: *VDBP* and COPD

Given the significant heterogeneity in the overall analyses, explorations were undertaken by subgroup analyses according to ethnicity, sample size, source of controls, matched condition, and diagnosis criteria of COPD, respectively (Table 2). Under the heterozygous genotype model, the association of *VDBP* gene rs7041 polymorphism with COPD was potentiated in Caucasians (OR: 0.16, 95% CI: 0.03 to 0.97, *p* = 0.046), in studies with matched patients and controls (OR: 0.19, 95% CI: 0.06 to 0.58, *p* = 0.004), in studies with doctor-diagnosed COPD (OR: 0.01, 95% CI: 0.00 to 0.10, *p* < 0.001), and adopting ATS criteria (OR: 0.44, 95% CI: 0.23 to 0.85, *p* = 0.014). Under the dominant model, COPD risk was significantly reduced in large studies (OR: 0.68, 95% CI: 0.48 to 0.95, *p* = 0.025), in studies enrolling population-based controls (OR: 0.58, 95% CI: 0.40 to 0.84, *p* = 0.004), in studies involving Caucasian populations (OR: 0.56, 95% CI: 0.38 to 0.83, *p* = 0.004), in studies with doctor-diagnosed COPD (OR: 0.35, 95% CI: 0.14 to 0.88, *p* = 0.025), and in ATS-criteria-based COPD (OR: 0.59, 95% CI: 0.36 to 0.97, *p* = 0.036). As shown in Table 2, the heterogeneity of each subgroup of race was still significant with a corresponding *I* ^2^ value of 78.1% in the Asian group and 81.8% in the Caucasian group, indicating that race was not the cause of heterogeneity under the heterozygous genotype model. Under the allele model and the homozygous genotype model, there was no noticeable significance across all subgroups.

**TABLE 2:** Subgroup analyses of *VDBP* gene rs7041 polymorphism with COPD risk under 4 models of inheritance.

Subgroup	Allele model (G vs*.* T)					Heterozygous model (GT vs*.* TT)				Homozygous model (GG vs*.* TT)				Dominant model (GG plus GT vs*.* TT)			
	Studies	OR	95% CI	*p*	*I* ^*2*^	OR	95% CI	*p*	*I* ^2^	OR	95% CI	*p*	*I* ^2^	OR	95% CI	*p*	*I* ^2^
**By sample size**																	
Total sample size <220	6	0.95	0.48 to 1.90	0.893	89.7%	0.35	0.14 to 0.91	0.072	80.1%	1.40	0.52 to 3.79	0.512	79.0%	0.63	0.27 to 1.46	0.283	86.6%
Total sample size ≥220	6	1.09	0.81 to 1.47	0.552	73.8%	0.53	0.27 to 1.03	0.062	78.0%	0.87	0.61 to 1.26	0.463	29.3%	0.68	0.48 to 0.95	0.025	54.8%
**By race**																	
Asian	7	0.96	0.57 to 1.59	0.861	88.5%	0.64	0.34 to 1.2	0.166	78.1%	1.29	0.57 to 2.89	0.544	76.8%	0.70	0.39 to 1.26	0.233	85.1%
Caucasian	4	1.29	0.86 to 1.94	0.224	74.0%	0.16	0.03 to 0.97	0.046	81.8%	0.94	0.60 to 1.48	0.788	31.6%	0.56	0.38 to 0.83	0.004	19.9%
Middle Eastern	1	0.74	0.48 to 1.15	0.180	NA	0.75	0.34 to 1.63	0.462	NA	0.55	0.23 to 1.33	0.186	NA	0.67	0.32 to 1.40	0.286	NA
**By match**																	
No	5	1.07	0.79 to 1.45	0.657	67.5%	0.88	0.51 to 1.52	0.644	76.3%	0.94	0.58 to 1.54	0.808	33.2%	0.88	0.57 to 1.35	0.552	72.9%
Yes	7	1.00	0.57 to 1.76	0.992	89.1%	0.19	0.06 to 0.58	0.004	75.6%	1.18	0.57 to 2.46	0.651	77.6%	0.53	0.28 to 1.02	0.058	78.1%
**Diagnosis**																	
ATS	7	0.93	0.58 to 1.49	0.762	87.8%	0.44	0.23 to 0.85	0.014	72.4%	0.95	0.47 to 1.94	0.894	77.5%	0.59	0.36 to 0.97	0.036	75.8%
Doctor	2	1.02	0.33 to 3.13	0.980	86.4%	0.01	0.00 to 0.10	<0.001	0.0%	1.06	0.60 to 1.86	0.839	0.0%	0.35	0.14 to 0.88	0.025	58.6%
GOLD	3	1.30	0.95 to 1.78	0.102	44.7%	1.20	0.88 to 1.64	0.260	3.8%	1.50	0.82 to 2.76	0.190	12.0%	1.32	0.87 to 2.01	0.195	33.5%
**Source of controls**																	
Hospital	8	0.95	0.63 to 1.45	0.819	86.2%	0.64	0.36 to 1.14	0.132	75.3%	1.17	0.60 to 2.28	0.640	74.8%	0.69	0.41 to 1.19	0.182	82.8%
Population	4	1.24	0.76 to 2.03	0.392	80.8%	0.17	0.03 to 1.02	0.052	82.6%	0.94	0.55 to 1.63	0.835	40.6%	0.58	0.40 to 0.84	0.004	23.6%

Abbreviations: COPD, chronic obstructive pulmonary disease; OR, odds ratio; 95% CI, 95% confidence interval; GOLD, Global Initiative for Chronic Obstructive Lung Disease; ATS, American Thoracic Society; *I* ^2^, inconsistency index; NA, not available.

### Meta-regression analyses: *VDBP* and COPD

Further explorations on the causes of heterogeneity were performed using meta-regression analyses via modeling age, male composition, BMI, percentage of smokers, smoking exposure (cigarette pack-years), FEV_1_, FEV_1_ predicted ratio, FVC, FVC predicted ratio, and FEV_1_/FVC in cases and controls under the four genetic models. However, there was no detectable significance for all factors (*p* > 0.05).

### Cumulative and sensitivity analyses: *VDBP* and COPD

The impact of the first published article on subsequent articles was not significant for the association between rs7041 polymorphism and COPD in cumulative analyses (Supplementary Figure S1). In addition, the impact of any single article on pooled estimates was also not significant in sensitivity analyses (Supplementary Figure S2).

### Publication bias: *VDBP* and COPD

**FIGURE 3:**
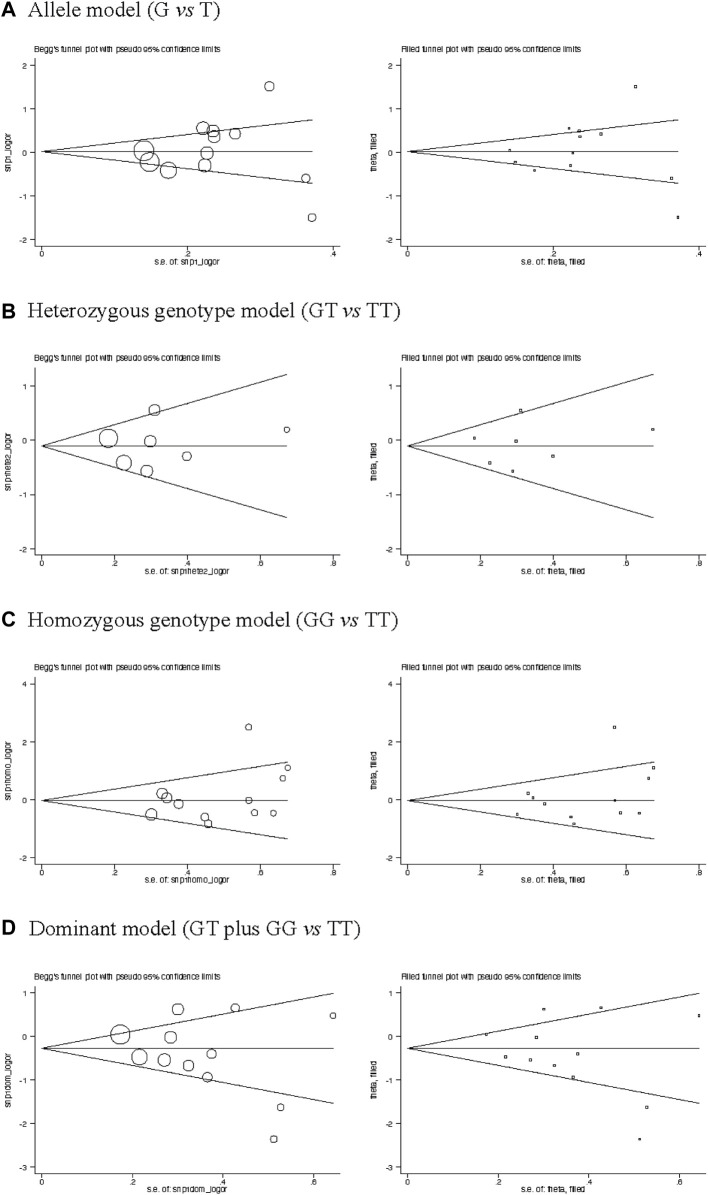
Funnel plots of *VDBP* gene rs7041 polymorphism with COPD under four models of inheritance. **(A)** Allele model (G vs. T), **(B)** Heterozygous genotype model (GT vs. TT), **(C)** Homozygous genotype model (GG vs. TT), **(D)** Dominant model (GT plus GG vs. TT).

The Begg’s funnel plots seemed symmetrical for the association of rs7041 polymorphism with COPD risk under the allele, homozygous genotype, heterozygous genotype, and dominant models (Figure 3). This symmetry was confirmed by the Egger tests, with a corresponding probability of 0.725, 0.869, 0.249, and 0.276, respectively, indicating a low likelihood of publication bias.

### 25OHD changes and rs7041 genotypes

**FIGURE 4:**
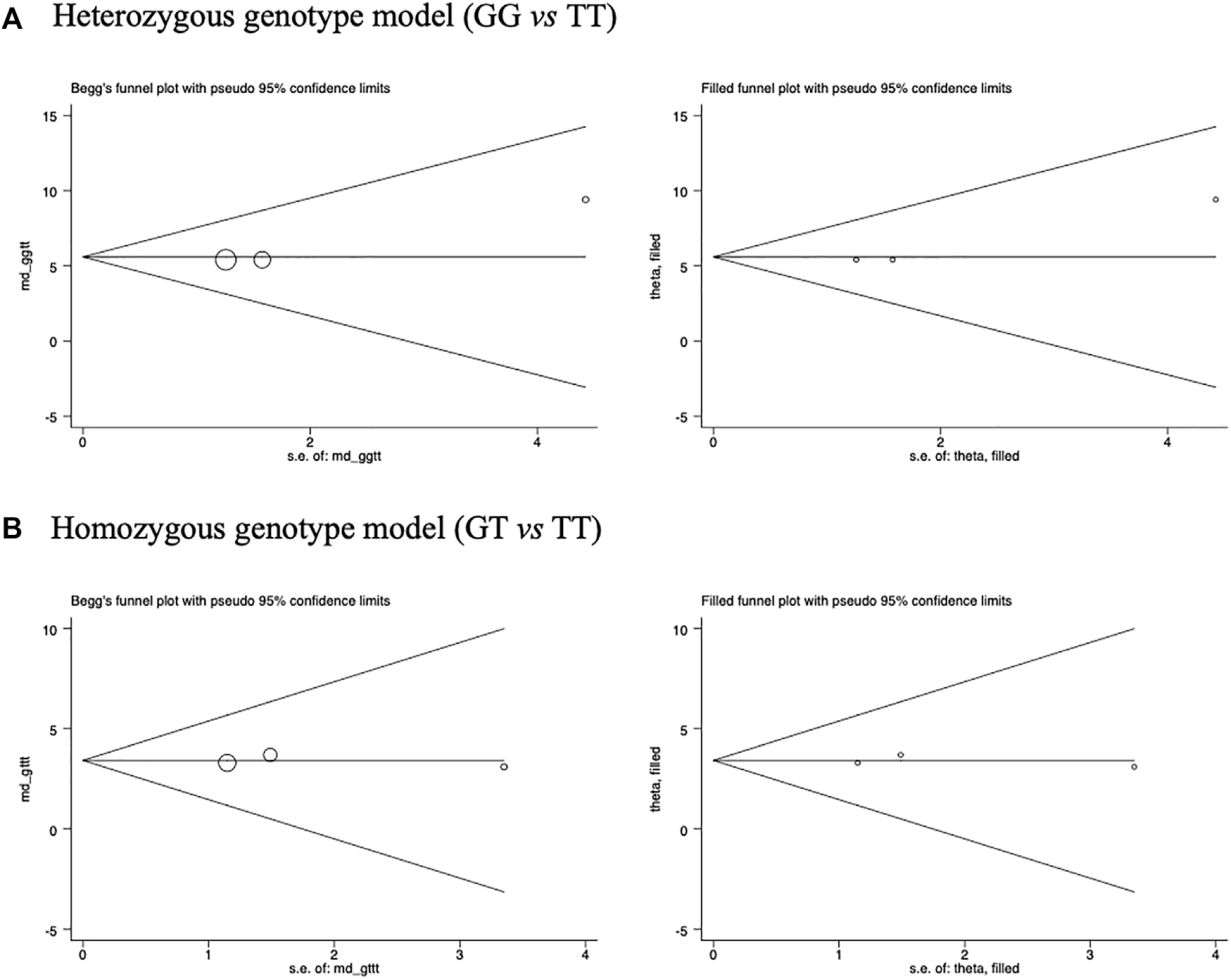
Funnel plots of the changes in 25OHD concentrations across rs7041 genotypes. **(A)** Homozygous genotype model (GG vs. TT), **(B)** Heterozygous genotype model (GT vs. TT).

Changes in circulating 25OHD concentrations across rs7041 genotypes were assessed. As shown in Figure 3, circulating 25OHD concentrations were significantly elevated in rs7041-GT genotype carriers (WMD: 0.32 ng/ml, 95% CI: 0.09 to 0.55, *p* = 0.006) and rs7041-GG genotype carriers (WMD: 0.58 ng/ml, 95% CI: 0.30 to 0.80, *p* < 0.001) compared to rs7041-TT genotype carriers, with low evidence of heterogeneity (*I* ^*2*^ = 22.2 and 1.4%, respectively), indicating no significant heterogeneity. In other words, race was not a significant cause of heterogeneity in our meta-analysis. The Begg’s funnel plots seemed symmetrical for the association of rs7041 polymorphism with the changes of 25OHD concentrations under homozygous genotype and heterozygous genotype models (Figure 4).

### Mendelian randomization analyses

**FIGURE 5:**
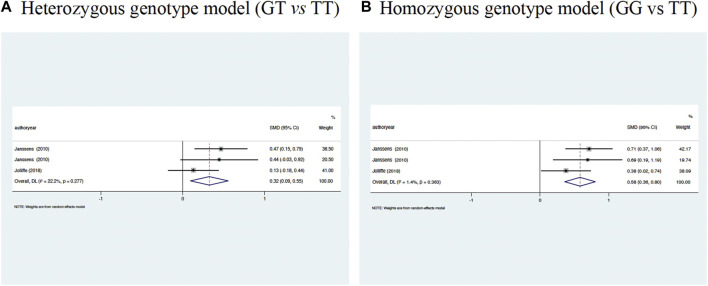
Circulating 25OHD changes across *VDBP* gene rs7041 genotypes. **(A)** Heterozygous genotype model (GT vs. TT), **(B)** Homozygous genotype model (GG vs. TT).

Analogous to a randomized controlled trial, Mendelian randomization is developed as a viable strategy to obtain unconfounded and unbiased estimates of causal relevance from observational data. To infer a causal relationship between exposure and disease outcome, two important prerequisites must be met: 1) the selected genes are significantly correlated with intermediate phenotypes or exposure; and 2) the selected genes are significantly correlated with the condition of the disease outcome (Sheehan et al., 2008). To apply R package’s “IVW” for Mendelian randomization analyses, we need the data consisting of the OR and 95% CI of rs7041 polymorphism across COPD patients and controls as well as the OR and 95% CI of circulating 25OHD changes across rs7041 polymorphism under the background of case control studies about COPD patients and controls as shown in Figure 2 and Figure 5, which show that the two important prerequisites mentioned above were both met properly. Specifically, the causal estimate was obtained by regression of the associations with the outcome on the associations with the risk factor, with the intercept set to zero and weights being the inverse variances of the associations with the outcome when the “IVW” package was applied (Burgess et al., 2013). With a single genetic variant, this was simply the ratio method (Burgess et al., 2013). Therefore, assuming a linear logistic relationship between circulating 25OHD concentrations and the risk of COPD, an inverse association (WMD: −2.07, 95% CI: −3.72 to −0.41, *p* = 0.015) was noted after using *VDBP* gene rs7041 polymorphism as an instrument for Mendelian randomization analyses.

## Discussion

In this meta-analysis, we aimed to evaluate the association of *VDBP* gene rs7041 polymorphism with the risk of COPD and circulating 25OHD changes. After integrating the results of 13 articles and 3,389 participants, we observed significant associations of this polymorphism with the risk of COPD and changes in circulating 25OHD concentrations. Importantly, under the rationale of Mendelian randomization, we found a causal relationship between genetically regulated 25OHD concentrations and COPD risk. To the best of our knowledge, this is the first meta-analysis to date that has interrogated the causality between circulating 25OHD and COPD in the literature.

The association of *VDBP* gene rs7041 polymorphism with COPD risk has been widely studied. Li et al. (2014) found that COPD patients were at high risk of vitamin D deficiency, and that carrying the rs7041-T allele had an impact on serum 25OHD concentrations that were closely related to COPD susceptibility. In contrast, Ishii et al. (2014) reported that distributions of rs7041 genotypes were comparable between COPD patients and controls. This inconsistency is likely due to inadequate sample size, patient selection, or lack of adjustment for confounders. To address this point, we performed subgroup analyses and found that the association between the rs7041-GT genotype and COPD risk was significant in populations of Caucasian origin and in studies with matched patients and controls. In view of this ethnic difference, we suggest building a candidate list of susceptible genes for COPD in each ethnic group.

Growing evidence shows that vitamin D is a promising biomarker in the development of COPD (Fu et al., 2021). The results of Fu et al. (2021) showed that vitamin D was inversely correlated with inflammatory signaling in patients with COPD, and that vitamin D may be a vital mediator of the progress of COPD in patients with low vitamin D levels. *VDBP* gene rs7041 polymorphism is responsible for the binding and transport of vitamin D analogs, as well as certain immune functions (Rozmus et al., 2020; Abdelaziz et al., 2021; Rozmus et al., 2022). In agreement with the findings of this meta-analysis, many studies have supported the close relationship between *VDBP* genetic alterations and circulating 25OHD concentrations. For instance, Alharazy et al. (2021) showed that the mutation of rs7041 polymorphism was associated with total 25OHD concentrations in postmenopausal women in Saudi Arabia, and the *post hoc* test indicated that total 25OHD concentrations were lower in carriers of the rs7041-TT genotype than carriers of the rs7041-GG genotype. In a separate study, Al-Daghri et al. (2019) reported that median 25OHD concentrations in carriers of the rs7041-GG genotype were significantly higher than in rs7041-TT genotype counterparts. In the present meta-analysis, we found that circulating 25OHD concentrations were significantly higher in carriers of the rs7041-GT genotype than those with rs7041-TT genotype and, importantly, that genetically regulated 25OHD in circulation played a causal role in the pathogenesis of COPD by means of the Mendelian randomization technique. Nevertheless, we concede that our findings are preliminary and that further independent validations are necessary.

Despite the clear strengths of this meta-analysis, including the large sample size and the adoption of the Mendelian randomization technique to infer causality between circulating 25OHD and COPD, several limitations should be acknowledged. First, caution should be taken when interpreting the relationship of circulating 25OHD concentrations with COPD because only two studies, with only 440 COPD patients and 152 controls, were available for analysis. Second, only articles published in the English language were retrieved, which might yield possible selection bias, even though our funnel plots and statistical tests revealed that this bias was unlikely. Third, although several subgroup analyses were conducted to explore potential heterogeneity, including analyses grouped by race, these subgroup analyses could not demonstrate that the target loci are conserved across different ancestries, and we only chose *VDBP* gene rs7041 polymorphism as an instrument for Mendelian randomization analysis, while other *VDBP* gene polymorphisms were not involved, thus limiting the interpretation of the pooled estimates.

In summary, we observed significant associations of *VDBP* gene rs7041 polymorphism with the risk of COPD and changes in circulating 25OHD concentrations. Importantly, under the rationale of Mendelian randomization, we found a causal relationship between genetically regulated 25OHD concentrations and COPD risk. Furthermore, our findings have highlighted the importance of employing the Mendelian randomization technique to pinpoint causal biomarkers and have provided clues regarding the causative pathways responsible for the biological regulation of 25OHD concentrations in COPD, thereby shedding new light in the physiology of this disease.

## Data Availability

The original contributions presented in the study are included in the article/Supplementary Material; further inquiries can be directed to the corresponding authors.
